# COVID-19: a modern trigger for Guillain-Barre syndrome, myasthenia gravis, and small fiber neuropathy

**DOI:** 10.3389/fnins.2023.1198327

**Published:** 2023-08-30

**Authors:** Francisco Gomez, Ashir Mehra, Erik Ensrud, Daniel Diedrich, Krzysztof Laudanski

**Affiliations:** ^1^Department of Neurology, University of Missouri, Columbia, MO, United States; ^2^Department of Anesthesiology and Perioperative Care, Mayo Clinic, Rochester, MN, United States

**Keywords:** COVID-19, SARS-CoV-2, peripheral neuropathy, Guillain-Barre, GBS, myasthenia gravis, small fiber neuropathy

## Abstract

COVID-19 infection has had a profound impact on society. During the initial phase of the pandemic, there were several suggestions that COVID-19 may lead to acute and protracted neurologic sequelae. For example, peripheral neuropathies exhibited distinctive features as compared to those observed in critical care illness. The peripheral nervous system, lacking the protection afforded by the blood–brain barrier, has been a particular site of sequelae and complications subsequent to COVID-19 infection, including Guillain-Barre syndrome, myasthenia gravis, and small fiber neuropathy. We will discuss these disorders in terms of their clinical manifestations, diagnosis, and treatment as well as the pathophysiology in relation to COVID-19.

## Introduction

Severe manifestations of COVID-19 may be partly accounted for by an autoimmune reaction mediated by a dysregulated network of circulating proinflammatory cytokines and inflammatory markers, including IL-1β, IL-6, IL-2, IL-8, IL-17, TNF-α, C-reactive protein, D-dimer, and antibodies (da Silva et al., 2021; Qin et al., 2022). It has been postulated that the resulting hyperinflammatory state causes endothelial dysfunction with increased vascular permeability, and hypercoagulability. These may progress to more severe complications such as acute respiratory distress syndrome and multi-organ failure (Ginikopoulou, 2022; Qin et al., 2022). Additionally, the inflammatory state may incite damage to the unprotected nerve fibers and prolonged resolution may result in ongoing exposure to non-specific inflammatory reactions. The emergence of autoimmunity can occur via numerous mechanisms; (a) if there is failure to suppress autoreactive clones (breakdown of immune tolerance measures) (b) if viral proteins that share an anatomical resemblance to innate proteins trigger an immune response (molecular mimicry) (c) if progressive infection leads to epitope diversification and thereby provoking an autoimmune response (Jovanova-Nesic and Shoenfeld, 2006; Morsy, 2020; Jacob et al., 2022). Interestingly, other evidence suggested autoreactive molecules resembling severe acute respiratory syndrome coronavirus-2 (SARS-CoV-2) like NCAM-1 were elevated (Laudanski et al., 2021). The present manuscript describes the pathogenesis, clinical presentation, and management of three neurological disorders in the setting of recent SARS-CoV-2; namely these include Guillain-Barre Syndrome (GBS), Myasthenia Gravis (MG), and Small Fiber Neuropathy (SFN).

Guillain-Barre Syndrome (GBS) and Myasthenia Gravis (MG) are recognized autoimmune illnesses. Likewise, because some cases of SFN are immune mediated, they can be triggered by COVID-19 as well (Zhou, 2019). Thus, these disorders of the peripheral nervous system may be caused or worsened by the dysregulated systemic immune response to COVID-19 infection or its aftermath. On the other side, if dysregulated response underlies post-COVID-19 peripheral neuropathies, immunomodulating strategies commonly employed in the treatment of neurological autoimmune diseases would ameliorate post-COVID-19 neurological sequelae.

## Methods

Search strategy and selection criteria: PubMed and Google Scholar searches were employed utilizing the following keywords: “COVID-19” “Sars-COVID-19” in combinations with “Peripheral Neuropathy,” “GBS,” “Guillain-Barre,” “MG,” and “SFN” was conducted for the years 2018–2022. Additional review articles explaining previously established pathophysiology for said diseases was included, dated prior to 2018.

Review articles and meta-analyses were included on rare occasions to provide readers with further details and references. Articles were evaluated for relevancy related to concomitant establishment of the above described neurologic and COVID diagnose by EE, AM, and FG. FG also served as the final arbiter for inclusion. Relevant references from these publications that focused on COVID-19 pathophysiology were also included.

## Discussion

### COVID-19 and Guillain Barre-syndrome

#### Definition

Guillain-Barré syndrome (GBS) comprises a gamut of autoimmune polyneuropathies varying in pathophysiology and symptoms (Fokke et al., 2013; Guidon and Amato, 2020). The hallmark clinical findings in these disorders are flaccid weakness and hyporeflexia (Shahrizaila et al., 2021). GBS can be broadly divided into demyelinating and axonal variants depending on the peripheral nerve site of autoimmune response (Shang et al., 2021).

#### Epidemiology

The incidence of pre-covid GBS is estimated at 100,000 new cases per year worldwide with regional variability. The incidence increases with age and is higher in men (Shahrizaila et al., 2021). A precipitating infection within 4 weeks often precedes GBS. Known associated viruses including Influenza A, Epstein–Barr, hepatitis E, and Zika have all been reported and well described (Shahrizaila et al., 2021; Shang et al., 2021). GBS associated COVID-19 cases have followed a similar epidemiological pattern, with older men, averaging 61 years old, being affected more frequently than women, at a nearly 2:1 ratio in case series (Pimentel et al., 2023). Similarly, a lag between the COVID-19 infection and GBS symptoms onset averages 14–19 days which is similar to previously described precipitating infections (Shahrizaila et al., 2021; Aladawi et al., 2022; Pimentel et al., 2023). These similarities indicate that the pathophysiology of GBS in the setting of recent COVID-19 is similar to GBS triggered by other infectious agents (Aladawi et al., 2022).

Reports have varied on the association between GBS and COVID-19, with no early conclusive evidence of an increased risk for GBS (Suh and Amato, 2021). Rather, a cohort study conducted in Britain found a decrease in GBS incidence during the pandemic, which the authors attributed to a generalized decrease in the incidence of precipitating infections due to the adopted lockdown measures (Keddie et al., 2021). Additionally, it is possible that GBS cases were under-reported during said period. Notwithstanding, the sheer number of reported cases of GBS in association with prior COVID-19 infection does suggest an association to the authors. However, most reports detailing the association between COVID-19 and GBS arose early in the epidemic (Abu-Rumeileh et al., 2020; Caress et al., 2020; Paterson et al., 2020; Toscano et al., 2020). Further assays have shown a possible slight increase, wherein a multi-center study involving 61 emergency departments in Spain found a slight increase in relative frequency of GBS among COVID (0.15‰) vs. non-COVID (0.02‰) patients (odds ratio [OR] = 6.30, 95% confidence interval [CI] = 3.18–12.5) The authors concluded that GBS is not often a debuting presentation for COVID infection (Fragiel et al., 2020).

Considering that GBS is an autoimmune disease secondary to a trigger that activates the immune system, it is unsurprising COVID-19 infection is linked to an increased risk of GBS (Kanou et al., 2022). Unfortunately, several of said reports were confounded by the concomitant use of experimental therapies for COVID-19 including steroids, antiviral medications as well as other sequelae of COVID-19 such as critical care illness neuropathy (FINSTERER et al., 2021). Furthermore, establishing a link is further complicated by the concomitant administration of vaccines which may trigger non-specific immune reactions with potential to impact the nervous system. In any case, the frequency of post-COVID-19 vaccine related GBS is lower than GBS provoked by COVID19 infection (Patone et al., 2021).

#### Pathogenesis

**Figure 1:**
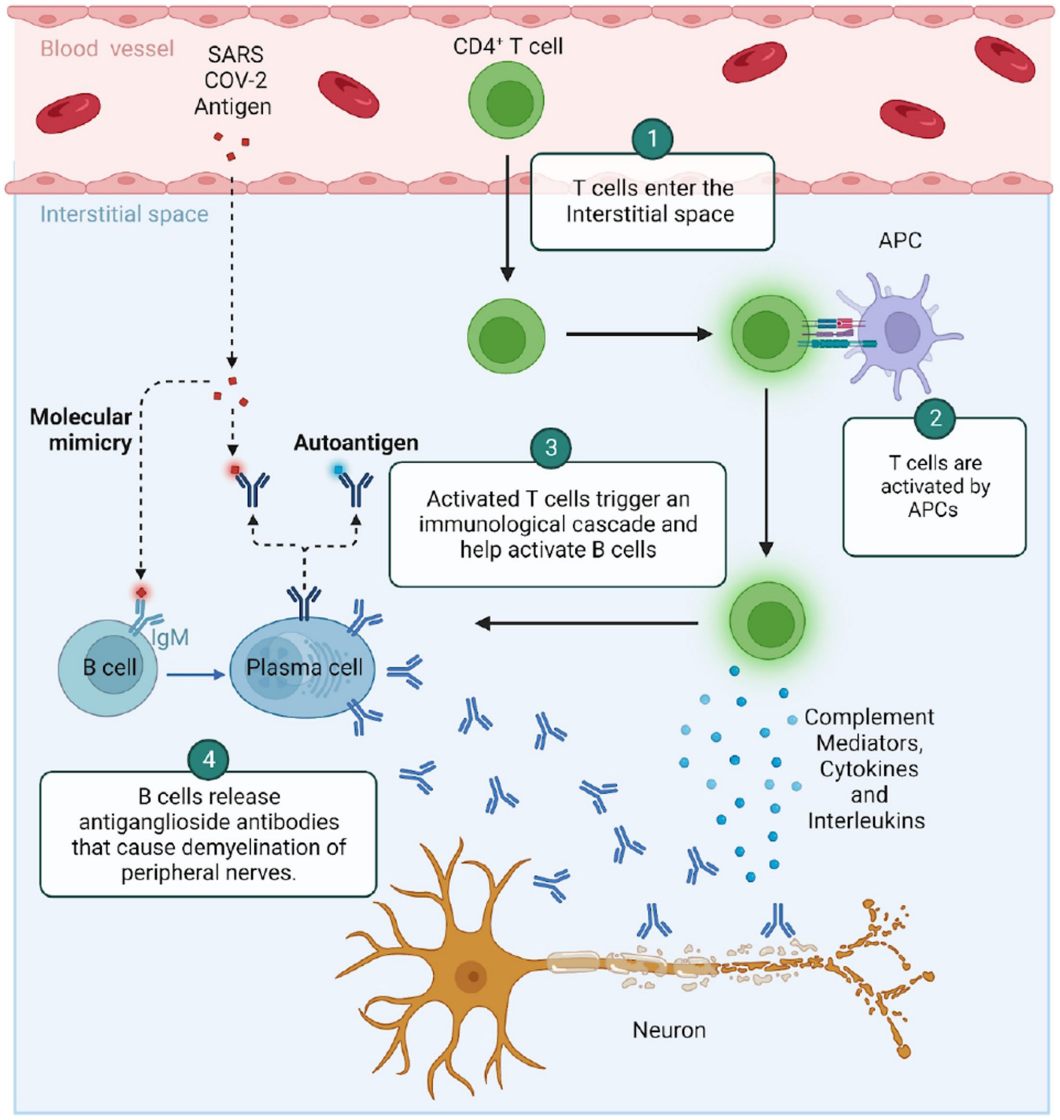
Suspected pathogenesis of Guillain-Barré syndrome in the setting of COVID-19.

Traditionally, GBS is thought to arise from molecular mimicry between offending (infectious, vaccine, drugs) agents and peripheral neuron gangliosides leading to the generation of anti-ganglioside antibodies (Guidon and Amato, 2020; Suh and Amato, 2021). It is important to note that various forms of GBS have unique pathogenic mechanisms. The most common form of GBS, Acute Inflammatory Demyelinating Polyneuropathy (AIDP) occurs because of T-cell mediated cytokine storm and does not routinely have detectable antibodies (Shang et al., 2021). Conversely, the axonal variants of GBS, namely Acute Motor Axonal Neuropathy (AMAN) and Acute Motor and Sensory Axonal Neuropathy (AMSAN) are associated with the traditional anti-ganglioside antibodies (Shang et al., 2021; Figure 1).

Known GBS specific auto-antibodies have been found in COVID-19-related GBS. Sporadic cases with positive auto-antibodies such as anti-GM1, GM2, GD1a, GD1b, GD3, GM1, GT1b or contactin have been found only rarely (Suh and Amato, 2021; Taga and Lauria, 2022). Further meta-analysis reported antiganglioside antibodies in merely 2% of cases, the most common being anti-GD1b IgG (Pimentel et al., 2023). *In vitro* assays have identified molecular similarities between COVID-19-encoded protein and host neuronal proteins raising the potential of autoimmune mimicry (da Silva et al., 2021), similarly, cross reactivity between COVID-19 neutralizing and neuronal epitopes has been reported (Kreye et al., 2020). *Per contra*, in-silico peptidome studies showed conflicting results (da Silva et al., 2021). One analysis identified molecular structural similarities at the molecular level between COVID-19 peptide sequences and adhesion molecules expressed by neurons and Schwann cells Another noted potential for molecular mimicry between COVID-19 and heat shock proteins-60 and -90 (Hsp) (Lucchese and Flöel, 2020; Shang et al., 2021). Both Hsp were linked to the emergence of GBS. An example of potential mimicry is a single viral open reading frame (ORF1) protein, a part being coded by SARS-CoV-2 genome that shares a sequence with human mono-ADP-ribosyltransferase (PARP14), with a 32% match suggesting structural mimicry (Keddie et al., 2021). These suggestions are not universal as another study found no homologies between viral membrane, spike or nucleocapsid COVID-19 encoded peptides and those in human neural tissue. Thus, the matter of whether a specific COVID-19 encoded protein is generally causative of GBS remains to be fully ascertained. It has been suggested that given para-infectious or a post-infectious symptomatic debut, the trigger for GBS associated with COVID may be overactivation of the systemic inflammatory response rather than specific epitope per se causative of molecular mimicry. These authors cite the abundance of circulating IL-6 and other inflammatory cytokines to be a more likely suspect (Ahmad et al., 2022). Another proposed etiopathogenesis suggests it is feasible that virus neurotropism for olfactory bulb cells with resulting inflammation and demyelination leads not only to described anosmia and dysgeusia but expression of hitherto unexposed epitopes, including GD1b (Fragiel et al., 2020).

There is also the potential that non-specific mimicry is induced via antibody activation. *Campylobacter jejuni*, is the most studied model in the axonal form of GBS with ample evidence supportive of molecular mimicry (Suh and Amato, 2021). In this model, B and T cells are activated by antigen presenting cells by processing the offending pathogen and selecting reactive T and B cells to produce antibodies via hypermutation mechanism. However, the fault in the system can cause B cells to produce antibodies that are avid for ganglioside antigens. These immunoglobulins bind proteins on Schwann cell Ranvier nodes triggering complement and attracting acquired immunity components. Subsequently, neuronal axolemma is damaged resulting in primary neuropathy.

Via independent and complementary mechanisms, co-activated T cells produce proinflammatory cytokines and chemokines that facilitate entry of macrophages into the neural tissue (Shang et al., 2021). Prior histologic examinations in AIDP have demonstrated neural T-cell and macrophage infiltration, as well as complement deposition in Schwann cells while acute motor axonal neuropathy (AMAN) variants exhibit primary macrophage-mediated axonal injury with scarce demyelination or T-cell infiltration (Shahrizaila et al., 2021). In COVID-19-related AIDP, one small histological series demonstrated no viral invasion but primarily CD68^+++^ histiocytes, often accompanied by cytotoxic CD8^++^ T-cells and less frequently helper CD4^+^ T cells. This process is often fueled by interferons (Suh et al., 2021). This leukocyte composition demonstrates an activated immune system with little control over its response. The outcome is a damage to Schwann cells with subsequent deterioration of the peripheral nerve function. In an interesting observation, perivascular inflammation was demonstrated in 67% of samples, with endoneurial infiltrates in only 11%. This may suggest a potential link between endothelial inflammation and peripheral nerve function but more definite studies are needed.

#### Clinical presentation

Classical Guillain-Barré syndrome comprises flaccid ascending limb weakness with hyporeflexia. Miller-Fisher Syndrome is a common variant consisting of hyporreflexia, accompanied by bilateral ophthalmoplegia, and ataxia. Other less common presentations include facial diplegia or pharyngeal-cervical-brachial paresis (Shahrizaila et al., 2021). Thus, GBS should be suspected in patients with rapidly progressive bilateral leg or arm paresis in the absence of CNS involvement. Concomitant distal paraesthesias or hypoesthesia are common in the sensorimotor variant (AIDP) (Leonhard et al., 2019). Concurrent respiratory paresis in GBS and COVID-19 infection necessitates early recognition given its rapidly progressive nature and potential tractability (Sriwastava et al., 2021).

COVID-19 related GBS infection most commonly presents with the classic sensorimotor variant, often accompanied by facial paresis. Electrophysiological testing showed a preponderance of demyelinating patterns (Aladawi et al., 2022). One single center study comparing 20 patients with COVID + GBS and GBS alone, those patients with concomitant COVID presented with statistically significant higher disability upon admission, higher incidence of cranial neuropathies and lower lymphocyte count (Ahmad et al., 2022). Rare variants such as the Pharyngo-cervico-brachial variant of GBS have been reported (Table 1; Randhawa et al., 2021).

**Table 1:** Reported symptomatology in COVID-19-related AIDP.

Symptoms	Aladawi, *n* = 99
Hyporreflexia	93% (*n* = 93)
Paraparesis	82% (*n* = 81)
Sensory symptoms	41% (*n* = 41)
Quadriparesis	65% (*n* = 64)
Facial Palsy	42% (*n* = 42)
Dysphagia	18% (*n* = 18)
Bulbar paresis	12% (*n* = 12)
Dysarthria	11% (*n* = 11)
Diplopia	11% (*n* = 11)
Ophthalmoplegia	11% (*n* = 11)
Ataxia	18% (*n* = 18)

Symptoms encountered in COVID-19-Related GBS (Aladawi et al., 2022). Of note 84/99 patients included by Aladawi reportedly had Brighton criteria for level 1–3 certainty.

Respiratory failure in GBS can be caused by a combination of respiratory muscle paresis, airway compromise or an inability to control secretions (Shang et al., 2021). COVID-19 related GBS cases have a similar presentation and are expectedly at risk for respiratory failure (Aladawi et al., 2022). In one meta-analysis involving 436 patients, respiratory muscle paresis was described in 18% of the study population wherein 10% progressed to frank respiratory failure necessitating endotracheal intubation and mechanical ventilation (Pimentel et al., 2023). Although this may make it appear that the incidence of respiratory failure in COVID-19 related GBS necessitating mechanical ventilation would appear slightly lower than the 30% as reported in pre-COVID-19 literature (Shang et al., 2021), it must be noted that authors Pimental et al. specifically note that an additional 54 of the reviewed 436 patients were admitted to ICU for unspecified reasons and may have had respiratory failure (Pimentel et al., 2023).

Autonomic failure is another severe complication of GBS, associated with increased mortality and length of ICU stay previously described in 3–38% of GBS patients (Chakraborty et al., 2019; Leonhard et al., 2019). One meta-analysis described dysautonomia in COVID-19 related GBS in addition to the following (with frequency); hypotension (6.9%), arrhythmias (6%), urinary retention or incontinence (5%), hypertension (4%), fecal incontinence or diarrhea (3%) (Pimentel et al., 2023).

In general, the mortality from GBS is estimated at 5% and complications from the disease are common, with up to 20% of patients unable to walk independently at 1 year (Shahrizaila et al., 2021). A recent meta analysis showed COVID-19-related GBS patients fared worse with 9% mortality and 22% showing residual paresis (Pimentel et al., 2023).

#### Diagnostics

The diagnosis of GBS by biomarkers alone remains difficult with numerous antibodies being described. Negative antibody testing does not rule out GBS (Leonhard et al., 2019). That being said, antibodies can be useful in distinguishing between variants such as Miller-Fisher Syndrome in which AntiGQ1b are positive in 90% of cases (Leonhard et al., 2019). Other variants have less specific associations, AIDP is associated with anti-LM1 and Gal-C, while AMAN is associated with Anti-GM1, GM2, GD1b, GT1b, GM3, GD1a, and GalNac-GD1a (Shang et al., 2021). Again, antiganglioside Ab have been found very rarely in COVID-19 related GBS cases (Pimentel et al., 2023). A systematic review conducted by Aladawi et al. (2022) demonstrated that only 14% of COVID-19 associated GBS had demonstratable antiganglioside antibodies. Magnetic Resonance Imaging (MRI) which demonstrates lumbar radicular enhancement with 83% sensitivity in the acute phase (Shahrizaila et al., 2021). In a small case series, Berciano et al., 2017 employed ultrasound and described C5–C7 cervical radicular enlargement. Improvements in those parameters may correlate with the clinical course (Berciano et al., 2017).

Therefore, diagnosis of GBS relies largely on clinical manifestations. The Brighton Criteria remain the most widely adopted, wherein cases are divided into levels of certainty 1 through 4. Level 1 confers the highest degree of certainty but necessitates positive Cerebrospinal fluid (CSF) or electrodiagnostic findings consistent with the disease.

##### Cerebrospinal fluid testing

Cerebrospinal fluid findings consistent with the disease include <50/μl cells and elevated protein levels, termed cyto-albumin dissociation (Fokke et al., 2013). However, CSF results may not be diagnostic in the early course of the disease and up to 50% of patients may exhibit normal findings in the first week, and 30% in the second (Leonhard et al., 2019).

##### Electrodiagnostic testing

Electrodiagnostic tests can be helpful in differentiating GBS variants, but can be falsely negative within the first week of symptoms (Leonhard et al., 2019) hence studies can be performed (Rajabally et al., 2014). Furthermore, EMG can distinguish different types of GBS. AMAN demonstrates reversible conduction failure and may occasionally show reduced compound muscle action potentials (Shang et al., 2021). AIDP exhibits slowed sensory motor nerve conductions, with early F wave abnormalities and later an increased distal response latency (Rajabally et al., 2014; Shang et al., 2021). A preponderance of demyelinating AIDP patterns was encountered in 77% of patients, followed by motor sensory axonal variants in 13%, and motor axonal variants in 10% (Aladawi et al., 2022).

This variant distribution is quite similar to that previously described in the literature; wherein AIDP reported 72%; of cases and AMAN 14–18% (Rajabally et al., 2014).

#### Treatment

The mainstay of GBS treatment is immunomodulation via primarily immunoglobulin removal by plasma exchange (PLEX) or increased degradation with intravenous immunoglobulins (IVIG). Both have shown nearly equal effectiveness (Leonhard et al., 2019), improving the speed of recovery but not necessarily disease progression (Shahrizaila et al., 2021).

#### Plasma exchange

Plasma exchange is an extracorporeal therapeutic technique where plasma is removed from whole blood via membrane filtration, centrifugation, or a combination of both (Gwathmey et al., 2014; Fernández-Zarzoso et al., 2019; Bauer et al., 2022). The patient then receives replacement fluid and cellular blood components. In general, PLEX allows for the removal of various pathogenic substances or molecules including autoantibodies, immune complexes, and toxins (Fernández-Zarzoso et al., 2019). It is believed that predominant benefit of PLEX in GBS is related to diminished titer of the autoantibodies and removal of a causative agent. Unfortunately, large fluid shifts during implementation of PLEX may cause hemodynamic instability. In GBS patients with dysautonomia, PLEX is more problematic as the autonomic system has an impaired ability to compensate for large fluid shifts and the therapy may lead to an increase in hypotensive events (Shahrizaila et al., 2021).

Plasma exchange is a mainstay of GBS treatment, as a Level I recommendation with grade A evidence (Fernández-Zarzoso et al., 2019; Bauer et al., 2022). Dosing recommendations vary between four and seven sessions dosed at 50 mL/kg every other day (Fernández-Zarzoso et al., 2019; Shahrizaila et al., 2021). This is theorized to be a consequence of the accumulation of newly synthesized antibodies (Melzer et al., 2016). Elevated necrosis factor alpha (TNF-α), interleukin-1 (IL-1), IL-6, levels have been reported in severe COVID-19 (Lu et al., 2021). PLEX may exert benefits via direct removal of proinflammatory cytokines (Ginikopoulou, 2022; Qin et al., 2022). One early study found a significantly decreased D-dimer, ferritin, CRP, IL-6 and procalcitonin in COVID-19 patients who underwent PLEX (Gucyetmez et al., 2020). Furthermore, it has been suggested that convalescent plasma used as the replacement solution could possibly enhance derived benefits (Ginikopoulou, 2022).

Multiple studies have found PLEX to be beneficial, or at minimum safe in SARS-Cov-2 infections (Khamis et al., 2020; Faqihi et al., 2021; Kamran et al., 2021; Cegolon et al., 2022). Thus, it would be reasonable to recommend PLEX in the setting of COVID-19-related GBS.

#### Intravenous immunoglobulins

Intravenous immunoglobulins is a blood product consisting of pooled healthy donor immunoglobulins with pleiotropic immuno-modulating and anti-inflammatory effects (Shang et al., 2021). While IVIG’s mechanism of action remains to be fully elucidated, several hypotheses have been described or proposed. These include increased antibody catabolism, blockade of autoantibody Fc tail region, complement protein scavenging and inhibition, and macrophage and mononuclear phagocyte inhibition (Norris et al., 2020). Antiganglioside antibody dimerization leading to decreased serum immunogenicity has been posited as an additional mechanism in GBS patients (Shang et al., 2021).

For GBS, daily administration at 2 g/kg over 5 days has shown efficacy (Leonhard et al., 2019; Shahrizaila et al., 2021). IVIG may be preferable to PLEX in patients with dysautonomia (Shahrizaila et al., 2021). Side effects of IVIG include anaphylaxis in patients with pre-existing IgA deficiency, aseptic meningitis, headache hypertension, pulmonary edema and dermatitis (Melzer et al., 2016). Hepatic dysfunction and thrombosis are less commonly encountered (Shahrizaila et al., 2021).

Numerous meta-analyses and case series have demonstrated that IVIG is safe to administer in COVID-19 patients (Cao et al., 2020; Xiang et al., 2021; Marcec et al., 2022).

#### COVID-19 Vaccination and Guillain Barre-Syndrome

Since 1976, vaccinations against viral infections have been linked to the development of GBS where an increase in cases was observed after a widespread vaccination program was undertaken in the US (Schonberger et al., 1979). Further studies suggested an increased incidence of one additional GBS case per 1 million influenza vaccinations (Leonhard et al., 2019). Thus far, one multicenter case series reported 9 cases of GBS following COVID-19 vaccination but the denominator is unclear (Karimi et al., 2021). A meta-analysis, including data from 17 countries, has reported a total of 88 cases of GBS. Of note, 63% of these patients were male, and neurological symptoms appeared 14 days post-vaccination in keeping with previously reported GBS epidemiology (Abolmaali et al., 2022). The Center for Disease Control did report an increased risk of GBS among adults who received the J&J/Janssen COVID-19 vaccination but not after Pfizer-BioNTech or Moderna COVID-19 vaccination (Centers for Disease Control and Prevention, 2019; Hanson et al., 2022). Specifically, Hanson et al. reported that the risk of developing GBS within 21 days of Ad.26.COV2.S (Janssen) vaccine was 32.4 per 100,000 person-years. Patients that received mRNA vaccines though showed a much lower rate of 1.3 per 100,000 person-years that was similar to the background (Hanson et al., 2022).

This data is not surprising given vaccines are by design immunogenic, a pathophysiological predisposition to GBS development is plausible and clinicians should be alert to developing symptoms in patients following recent COVID-19 vaccinations. The most important message is that the benefits of vaccine administration continue to outweigh risks in terms of overall mortality and GBS incidence (Abolmaali et al., 2022). Recent meta-analysis including 48 publications including 2,110,441,600 participants revealed COVID vaccine related GBS at a rate of 3.09 per 1 million people within 6 weeks of vaccination, higher to that of the influenza vaccine (Finsterer et al., 2022).

### COVID-19 and myasthenia gravis

#### Definition

Myasthenia gravis is an autoimmune disorder caused by antibodies targeting components of the neuromuscular junction, most commonly postsynaptic acetylcholine receptors leading to paresis (Farmakidis et al., 2018).

#### Epidemiology

Myasthenia gravis is the most prevalent neuromuscular junction disorder (Gilhus and Verschuuren, 2015; Farmakidis et al., 2018; Bubuioc et al., 2021; Punga et al., 2022). MG has been increasing with an annual incidence in adults estimated to be 10–29/1,000,000 with a prevalence ranging between 100 and 350/1,000,000. Between the ages of 15–64, it is more common in women at a 2:1 ratio, whereas late-onset myasthenia after age 64 has a higher incidence in men (Gilhus and Verschuuren, 2015). A genetic predisposition has been described, wherein siblings or first-degree relatives exhibit a 4.5% increase in risk for developing MG (Melzer et al., 2016). Additionally, chronic immunosuppression or treatment with multiple drugs of this type has been described as a risk factor for the development of COVID-19 or a more severe course (Sanders et al., 2016; Guidon and Amato, 2020).

#### Pathogenesis

Myasthenia gravis is caused by antibodies binding the neuromuscular junction (NMJ) epitopes within the postsynaptic membrane (Bubuioc et al., 2021). The acetylcholine receptor (AChR) is the most commonly targeted (Gilhus and Verschuuren, 2015). NMJ physiopathology in these cases has been well documented; synapses are impaired via receptor blockage, increased internalization hence decreased receptor availability, and complement deposition leading to distortion of the endplate thus widening of the synaptic cleft. AChR antibody levels correlate with disease severity (Melzer et al., 2016). Other recognized causative antibodies include muscle-specific kinase (MUSK), lipoprotein-related protein 4 (LRP4), agrin, titin, and ryanodine (Bubuioc et al., 2021). The prevalence of said antibodies in one review has been reported as AChR in 80% of patients, MUSK in 4% and LRP4 in 2%, the remaining 5% remaining seronegative (Gilhus and Verschuuren, 2015). Geographic variations have been reported (Punga et al., 2022).

**Figure 2:**
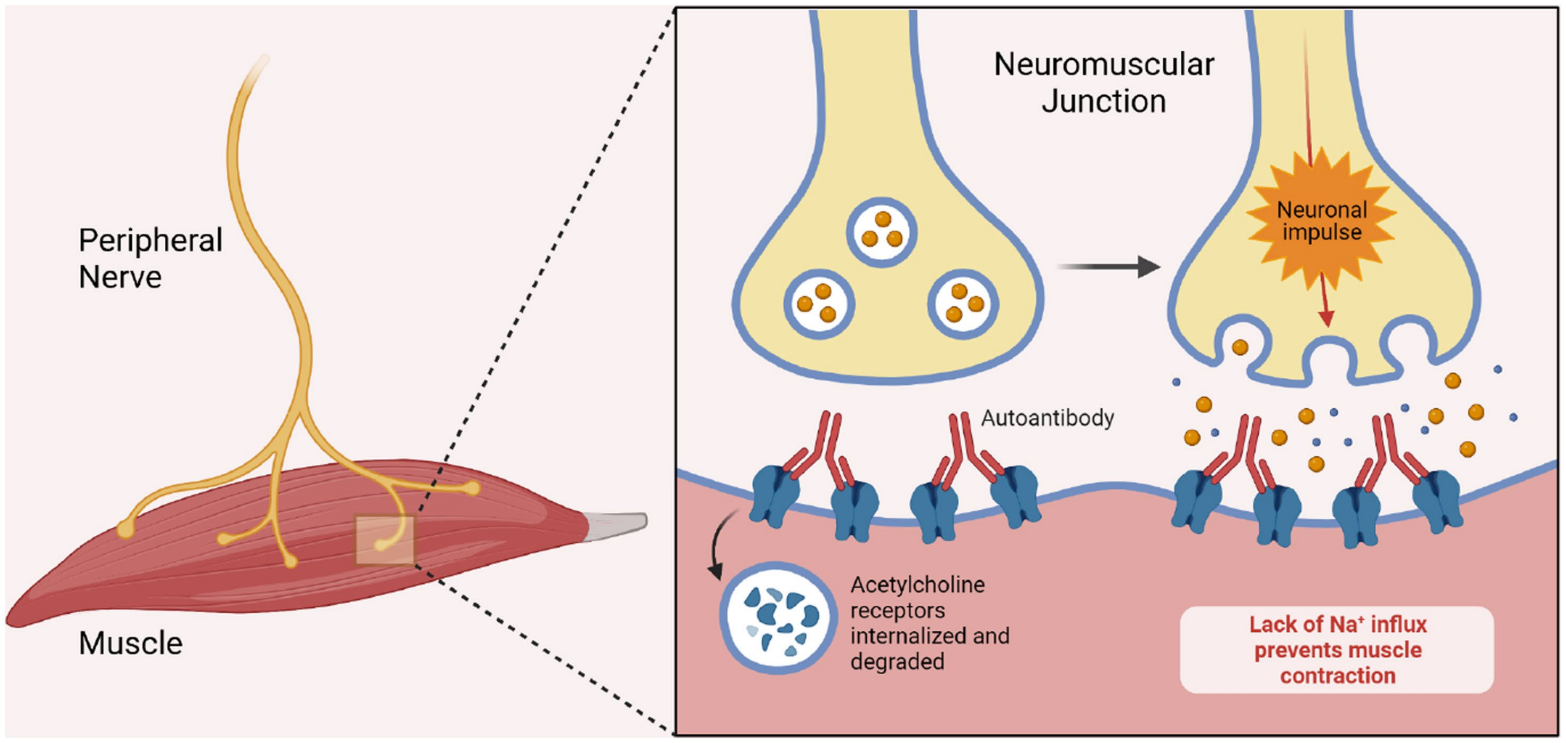
Pathogenesis of myasthenia gravis. The image demonstrates the pathogenesis of myasthenia gravis in the setting of ACh Receptor blocking antibodies. The antibodies bind to the post-synaptic acetylcholine receptors and thereby prevent depolarization of the muscular membrane. Biorender.com software.

A thymoma is associated with myasthenia gravis in 10–15% of cases (Melzer et al., 2016) wherein AChR auto reactive T-cells escape physiological surveillance and are released, subsequently activating B-cells. Thus, mediastinal imaging is recommended in all patients with this disease (Gilhus and Verschuuren, 2015; Punga et al., 2022). AChR expression by thymic epithelial cells may be incited by a viral infection via cytokine and receptor signaling (Gilhus and Verschuuren, 2015). MG subsequent to a viral infection has been previously reported following Epstein–Barr and Varicella-Zoster infections (Shah et al., 2022). Thus, unsurprisingly, new onset MG after SARS-Cov-2 infection has been reported with patients commonly testing positive for AChR Ab in the setting of ocular and bulbar symptoms (Huber et al., 2020; Restivo et al., 2020; Sriwastava et al., 2020; Assini et al., 2021; Essajee et al., 2021; Karimi et al., 2021). And although cases with positive MUSK antibodies have been documented as well, these appear to be less common (Figure 2) (Assini et al., 2021).

#### Clinical presentation

##### Generalized myasthenia

The most common symptoms in MG include fluctuating paresis, and muscle fatigability which is often progressive throughout the day (Gilhus and Verschuuren, 2015; Melzer et al., 2016; Bubuioc et al., 2021). Up to 60% of patients present with ptosis or diplopia, or a combination thereof (Gilhus and Verschuuren, 2015). Generalized myasthenia is described as paresis affecting any muscle groups beyond the ocular muscles. Weakness is most commonly found within bulbar or proximal limb muscle groups (Melzer et al., 2016). Bulbar weakness comprises dysphagia, dysphonia, difficulty chewing and dysphagia (Punga et al., 2022).

##### Ocular myasthenia

Ocular myasthenia is defined as paresis limited to the extraocular muscles leading to diplopia or ptosis and comprises 10–20% of cases (Melzer et al., 2016; Sanders et al., 2016). Ocular myasthenia is more often seen in patients with AChR antibodies, MUSK+ cases have been described, but are much rarer (Gilhus and Verschuuren, 2015).

##### Myasthenic crisis

A myasthenic crisis is defined as a rapid life-threatening exacerbation leading to respiratory failure (Sanders et al., 2016; Nelke et al., 2022) Respiratory failure can occur from a loss of airway protection or an inability to clear secretions both of which can occur from bulbar weakness. Respiratory failure can also occur from diaphragmatic paralysis and may affect up to 15% of MG patients (Punga et al., 2022). Infections are a common trigger for myasthenic crisis (Anand et al., 2020; Tugasworo et al., 2022) and associated with worse outcomes (Nelke et al., 2022). Previously used therapies that were used inappropriately to treat COVID-19, without solid evidence of efficacy, including HCQ and Azithromycin may actually worsen NMJ transmission (Qin et al., 2022). A few publications have reported MG following a SARS-CoV-2 infection and include a small retrospective case series involving 8 patients where MG exacerbation was attributed to a SARS-CoV-2 (Rodrigues et al., 2022). One particular case series reported myasthenic crisis necessitating rescue therapy in 36/91 (40%) of MG patients following COVID-19 (Muppidi et al., 2020). Another case series found more disturbing results with a high mortality (80%) in MG patients whom contracted COVID-19 (Lupica et al., 2022). These results could be attributed to the combined and synergistic effect of critical care illness and COVID-19 pathology.

#### Diagnostics

AChR antibodies, specifically the binding and modulating varieties, are highly specific for myasthenia gravis, and a positive assay in patients with muscle weakness is considered pathognomonic to the point of obviating electrodiagnostic tests (Gilhus and Verschuuren, 2015). Other detectable antibodies are described under the pathology section but are less common or reliable.

##### Electrodiagnostic

Electrodiagnostic tests continue to be of value, especially in seronegative patients. Single fiber EMG is the most sensitive test, while low-frequency repetitive nerve stimulation is often considered the first line procedure in patients with synaptic transmission failure (Gilhus and Verschuuren, 2015; Punga et al., 2022). Low-frequency repetitive nerve stimulation (3 Hz) is considered positive when there is a response amplitude decrease at a minimum of 6–10% between the first and fourth elicited compound motor action potentials (Punga et al., 2022). Single fiber electromyography measures muscle jitter, defined as the time interval variation between action potentials, which is increased in MG (Melzer et al., 2016; Punga et al., 2022).

#### Treatment

##### Maintenance treatment

Myasthenia gravis management involves enhancement of acetylcholine availability within the NMJ via inhibition of cholinesterase enzymes, or immunosuppression/immunomodulation (Farmakidis et al., 2018). Pyridostigmine, an acetylcholinesterase inhibitor (AChEI) is considered the first line treatment that also improves electrodiagnostic measures (Melzer et al., 2016). In an early randomized trial where the treatment arm (94/188) received pyridostigmine vs. placebo, the initial results demonstrated a tendency towards improved survival of 11.7%. Unfortunately, this trial was halted early due to lack of recruitment (Fragoso-Saavedra et al., 2022). Acetylcholinesterase inhibitors along with corticosteroids or azathioprine are considered first-line treatments (Melzer et al., 2016; Sanders et al., 2016). A variety of immunosuppressive therapies are employed as second line, or steroid sparing agents (Sanders et al., 2016). Notably, patients with ocular myasthenia exhibit a reduced rate of progression to the generalized form with the management strategy (Melzer et al., 2016). MUSK+ patients tend to respond less to acetylcholinesterase inhibitors and IVIG (Melzer et al., 2016; Sanders et al., 2016). However, commonly utilized immunosuppressants may influence COVID-19 outcomes (Rodrigues et al., 2022) (Table 2).

**Table 2:** Effects of immunosuppressants on COVID-19 mortality, note that some of this data was derived from Rheumatology patients and not exclusive to neuromuscular complications.

Treatment	Mechanism of action	Effect on COVID-19
Corticosteroids (Melzer et al., 2016; Farmakidis et al., 2018; Anand et al., 2020; Korsukewitz et al., 2020; Rodrigues et al., 2022)	Inhibit cytokine response, leukocyte recruitment. T-cell activation and differentiation suppressors	Increased mortality at higher doses
Azathioprine (Melzer et al., 2016; Farmakidis et al., 2018; Anand et al., 2020; Korsukewitz et al., 2020; Strangfeld et al., 2021; Rodrigues et al., 2022)	Purine synthesis suppressant. Inhibits cellular replication, and lymphocyte function	Possibly increased mortality
Cyclophosphamide (Melzer et al., 2016; Farmakidis et al., 2018; Korsukewitz et al., 2020; Strangfeld et al., 2021)	Cytotoxic guanine alkylating agent. Inhibits cell replicating by forming DNA-cross bonds. Marrow suppressant, inhibits B- and T-cells	Increased mortality
Cyclosporine (Farmakidis et al., 2018; Strangfeld et al., 2021; Rodrigues et al., 2022)	Calcineurin activation inhibitor, suppresses IL-2 and IFN-γ, Inhibits T-helper cell activation.	Did not affect outcomes
Eculizumab (Melzer et al., 2016; Farmakidis et al., 2018; Diurno et al., 2020; Korsukewitz et al., 2020; Mimori et al., 2022)	Monoclonal C5 complement inhibitor	Potentially beneficial
Tocilizumab (Anand et al., 2020; Korsukewitz et al., 2020; Rodrigues et al., 2022)	Monoclonal IL-6 inhibitor	Potentially beneficial*
Intravenous Immunoglobulins IVIG (Melzer et al., 2016; Farmakidis et al., 2018; Anand et al., 2020; Korsukewitz et al., 2020; Xiang et al., 2021; Rodrigues et al., 2022)	Inhibits macrophage Fc receptor expression, cytokine synthesis antibody production and complement activation.	Potentially beneficial
Mycophenolate (Melzer et al., 2016; Farmakidis et al., 2018; Anand et al., 2020; Korsukewitz et al., 2020; Strangfeld et al., 2021; Rodrigues et al., 2022)	Guanosine (purine) synthesis inhibitor	Possibly increased mortality
Methotrexate (Melzer et al., 2016; Farmakidis et al., 2018; Korsukewitz et al., 2020; Strangfeld et al., 2021; Rodrigues et al., 2022)	Dihydrofolate reductase inhibitor, thus inhibiting nucleotide synthesis.	No effect
Rituximab (Melzer et al., 2016; Farmakidis et al., 2018; Korsukewitz et al., 2020; Strangfeld et al., 2021; Rodrigues et al., 2022)	Recombinant antibody targeting CD-20+ B-cells.	Increased mortality
Plasma Exchange (Melzer et al., 2016; Farmakidis et al., 2018; Korsukewitz et al., 2020; Rodrigues et al., 2022)	Removal of autoimmune antibodies and cytokines	Potentially beneficial
Tacrolimus (Melzer et al., 2016; Strangfeld et al., 2021)	Calcineurin inhibitor, decreasing antigen-specific lymphocyte activation	Increased mortality

* Very limited data for tocilizumab as it is considered to be an experimental therapy for MG (Melzer et al., 2016; Farmakidis et al., 2018; Anand et al., 2020; Diurno et al., 2020; Korsukewitz et al., 2020; Strangfeld et al., 2021; Xiang et al., 2021; Mimori et al., 2022; Rodrigues et al., 2022).

Some immunosuppressive therapies utilized for the management of myasthenia have shown possible dual benefits. Tocilizumab, an IL-6 inhibiting monoclonal antibody indicated for treatment of severe COVID-19 has shown safety and efficacy in two previously refractory MG patients, and safety in a third patient in a small case series (Anand et al., 2020). A meta-analysis including 3,924 patients of which 433 received tocilizumab showed promising results. The treatment arm exhibited a lower adjusted mortality risk of 27.5% vs. 37.1% (95% CI, 21.2–33.8 and 95% CI, 35.5–38.7%, respectively) (Gupta et al., 2021). That being said, Tocilizumab is not yet approved for use in MG and it is still considered an experimental therapy.

Eculizumab, a monoclonal antibody directed at the complement attack complex, has demonstrated a benefit in the treatment of refractory MG in early trials (Melzer et al., 2016). Similarly, one small cohort study which included 10 patients in the eculizumab arm found the treatment to be safe and well tolerated in severe COVID-19 patients preventing them from being treated with advanced respiratory support, as well as noted improvement in respiratory distress and inflammatory markers. Moreover, the authors concluded the treatment arm tended towards decreased in-hospital mortality or respiratory sequelae (Ruggenenti et al., 2021).

##### Crisis treatment

Plasma exchange and IVIg are the mainstay of rescue management in myasthenic crisis (Melzer et al., 2016; Sanders et al., 2016). There is some data to suggest PLEX may exhibit quicker effect onset (Županić, 2021), but the guidelines do not strongly recommend one treatment over the other in the general MG population. Safety profile and effects for these treatments in the treatment of COVID-19 is as aforementioned.

#### COVID-19 vaccination and myasthenia gravis

Vaccinations are generally recommended for MG patients, including COVID-19. Non-live formulations may be preferable given common concurrent immunosuppressive treatments. Prior trials regarding seasonal influenza vaccines showed safety in MG (Županić, 2021). One retrospective case series evaluated 22 MG patients receiving inactivated or recombinant vaccines, 77% were on chronic immunomodulators. In total, two patients reported mild worsening symptoms, treated successfully with pyridostigmine (Ruan et al., 2021). Another study which included 53 MG patients receiving vaccinations showed similar results wherein the measured myasthenia gravis activities of daily living score was unaffected in 58.5%, improved in 15% and demonstrated worsening symptoms in 28.3%, independent of vaccine formulation, prior antibody titers, or MG variant (Lupica et al., 2022). Yet another case series found MG symptomatic decline after COVID-19 vaccination in 7.7% of 104 included cases, mostly mild (Farina et al., 2022).

In one multinational retrospective study involving COVID vaccinations and immune mediated disease, a total of 2 *de novo* myasthenia gravis cases occurred, both after the second dose of BNT162b2 vaccine, with one case described as severe (Watad et al., 2021). At the time of publication, a mere 6 cases of COVID-19 vaccine related MG have been reported (Lee et al., 2022; Sansone and Bonifati, 2022) with one of these patients presenting with myasthenic crisis (Sansone and Bonifati, 2022). Given the relative infrequency and mild symptomatology of adverse reactions following COVID-19 vaccine in MG patients, and the lack of robust information to infer association, vaccination is recommended in this patient population (Shah et al., 2022). Cases of wherein varying the administered vaccine type and immunomodulatory therapy resulted in a satisfactory rise in titers in patients with known MG (Sansone and Bonifati, 2022).

### COVID-19 and small fiber neuropathy

#### Definition

Small fiber neuropathy is an umbrella term comprising a varied group of disorders involving the peripheral thinly myelinated Aδ fibers and unmyelinated C nerve fibers (Zhou, 2019; Devigili et al., 2020). The pathophysiology of this disorder is unclear and appears to have multiple etiologies (Zhou, 2019). The symptom common to all is neuropathic pain (Strangfeld et al., 2021) and autonomic symptoms are a common finding (Sène, 2018; Devigili et al., 2020).

#### Epidemiology

Given protean symptoms and various causes, varying reports on epidemiological data are unsurprising. One study in Olmsted county, Minnesota United States reported an incidence of 1.3/100,000 which increased during the study period (Johnson et al., 2021). Reports on prevalence have ranged between 13 and 53 per 100,000 in the Netherlands and United States, respectively with conflicting data on predilection for men or women (Peters et al., 2013; Johnson et al., 2021). SFN is likely underdiagnosed leading to an underestimation of the true incidence and prevalence (Farhad, 2019). Exacerbations or, more importantly, *de novo* cases of SNF manifesting as COVID-19 sequelae have been reported and described as “not uncommon” (Abrams et al., 2021; Shouman et al., 2021).

#### Pathogenesis

The term “Small Fiber” refers to small somatosensory fibers, which mediate pinprick and thermal sensations, and autonomic C fibers, which innervate the smooth muscles of blood vessels, gastrointestinal track and genitourinary tract (Zhou, 2019). Thus, symptomatology comprises primarily dysesthesias or dysautonomia, respectively. SFN can be classified by pattern of involvement; length-dependent debuting commonly with distal sensory symptoms, non-length-dependent neuropathy with patchy involvement, or neuropathy multiplex or monoplex (Devigili et al., 2020). The most common variant is length dependent neuropathy (Zhou, 2019) as seen in Diabetes Mellitus, and is thought to account for 4.5–31% of cases (Sène, 2018; Farhad, 2019).

Small Fiber Neuropathy has been broadly organized into etiological categories which include: metabolic, inflammatory, toxic, infectious, genetic or idiopathic (Sène, 2018; Johnson et al., 2021). Specific diseases associated with autoimmune SFN include systemic lupus erythematosus, Sjogren’s syndrome, sarcoidosis or paraneoplastic syndromes (Shoenfeld et al., 2020). SFN and fibromyalgia have been linked to autoimmune processes (Oaklander and Nolano, 2019). Given an observed delayed symptomatic debut measured in weeks, some authors have postulated a postinfectious autoimmune injury mechanism for SFN subsequent to COVID-19 cases (Burakgazi, 2022). Yet another case series found 13 patients debuting with new onset paresthesias after COVID infection, wherein 6 had SFN confirmed via skin biopsy. Authors concluded SFN may underlie the paresthesias associated with so called “long-haul” COVID (Abrams et al., 2021), which these authors consider a neurologicsequelae.

#### Clinical presentation

A majority of patients do not self-report SFN symptoms as disabling, but quality of life can be severely decreased (Johnson et al., 2021). Dysautonomia results from autonomic C-fiber dysfunction and can affect several organ systems (Zhou, 2019). Gastrointestinal involvement may lead to chronic diarrhea or constipation, gastroparesis, pseudo-obstruction or fecal incontinence. Genitourinary involvement may manifest as dysuria, incontinence or impotence. Exocrine dysfunction of the sweat, salivary and lacrimal glands may also be encountered. Ocular manifestations may manifest as impaired accommodation, or photosensitivity (Sène, 2018).

Sensory symptoms can be described as negative or positive, the latter more commonly encountered (Devigili et al., 2020). Negative symptoms comprise decreased perception of stimuli while positive symptoms comprise perceived sensation disproportionate to or in the absence of stimuli. Sensory symptoms are most common in length-dependent SFN. Patients often present with sharp pain in the affected area, characterized as burning, lancinating or akin to an electrical discharge. Hyperalgesia and allodynia have been reported as well leading to discomfort with footwear or sheets (Zhou, 2019; Devigili et al., 2020). A squeezing sensation, coldness, or pruritus within the affected areas have been reported as well (Zhou, 2019). Positive symptoms may worsen at night time (Farhad, 2019; Devigili et al., 2020). Negative symptoms include hypoesthesia, as well as thermal perception and nociception (Devigili et al., 2020). Muscle strength would be preserved, as these functions are exerted by large nerve fibers (Zhou, 2019).

Cardiovagal dysfunction may be seen in up to 64% of SFN patients (Blackmore and Siddiqi, 2017). Signs and symptoms of cardiovascular dysautonomia include blood pressure lability including orthostatic hypotension, arrhythmias and sinus bradycardia or tachycardia (Sène, 2018). SFN patients may be at higher risk of myocardial infarctions with study finding an incidence of 46% vs. 27% in controls (*p* < 0.0001) (Johnson et al., 2021).

Neurological symptoms consistent with SFN following severe SARS-CoV-2 infection have been reported (Abrams et al., 2021). Furthermore, there is limited literature that has linked small fiber neuropathy to chronic fatigue syndrome (Shoenfeld et al., 2020). In view of previously described autoimmune etiologies and the fact that numerous SFN patients report a prior viral infection (Farhad, 2019), an autoimmune etiology to COVID-19-related SFN and a link between SFN and reported sequelae is possible.

An initial case report described a 64-year-old woman who developed a new painful SFN with concomitant fatigue, orthostatic dizziness, and urinary incontinence 2 weeks after COVID-19. The clinical condition improved with empiric IVIG (Novak, 2020) and the authors suggested a link with an autoimmune cause. Further case reports found 2 cases of length dependent neuropathy responding to pregabalin and duloxetine, respectively (Burakgazi, 2022).

Another single center’s retrospective review identified 27 patients with autonomic dysfunction subsequent to SARS-CoV-2 infection. Reported symptoms included lightheadedness (93%), orthostatic headache (22%), syncope (11%), hyperhidrosis (11%), and burning pain (11%). An abnormal sweat test was found in 36%, and cardiovagal dysfunction in 27% (Shouman et al., 2021).

Another case series included 13 patients with new onset symptoms after COVID-19. The authors took efforts to exclude confounding causes by testing HbA1c, antinuclear antibodies, vitamin B12, thyroid stimulating hormone and free T4, and performed serum immunofixation testing. Furthermore, none exhibited large fiber involvement in nerve conduction studies or electromyography. Biopsy confirmed SFN in 46% of cases. Painful paresthesias followed a length dependent distribution in 54% and a multifocal patchy distribution in the remaining 46% while orthostasis was also noted in 46% of the study population. The authors noted that although the study was likely underpowered, an association could be inferred (Abrams et al., 2021). A third case series involving 17 patients presenting after COVID-19 with no identified systemic or immune risk factors, SFN was confirmed in 6 via skin biopsy (Oaklander et al., 2022). While the data is limited, there exists a possible autoimmune etiology to COVID-19-related SFN and a link between SFN and reported sequelae.

#### Diagnostics

Small fiber neuropathy has for a long time been a clinical diagnosis based on the symptoms previously described. Allodynia with pinprick testing may be present evaluation (Blackmore and Siddiqi, 2017). Since deep tendon reflexes are mediated by large muscle fibers, hyporeflexia would not be expected (Zhou, 2019). Electrodiagnostic testing via nerve conduction studies is normal given this test does not measure the function of small fibers (Abrams et al., 2021). It should be noted that altered nerve conduction studies do not rule out SFN, but rule in further large fiber neuropathy as both pathologies can coexist (Sopacua et al., 2019). Thus, skin biopsy to evaluate nerve fiber density is considered by some authors to be the gold standard (Zhou, 2019). That being said, it must be noted that skin biopsy findings must be interpreted within the right clinical context and often in conjunction with already-established clinical criteria.

Diagnostic criteria for small fiber neuropathy have been proposed previously (Tesfaye et al., 2010; Blackmore and Siddiqi, 2017) However, established criteria may be biased towards the detection of length-dependent SFN as opposed to non-length dependent forms of the SFN. For example, the criteria proposed by Blackmore et al. include length dependent dysesthesias and abnormal pinprick sensation, altered pain or heat perception in addition to dysautonomia as tallied via quantitative sudomotor reflexes or abnormal heart rate variability testing (Blackmore and Siddiqi, 2017).

#### Treatment

The mainstay of SFN treatment comprises the identification and abatement of potential underlying causes. However, heterogeneity of the potential causes makes this an aspirational target. Symptom management includes gabapentin or pregabalin as well as antidepressants of the tricyclic and serotonin/norepinephrine uptake inhibitor variety as first line (Zhou, 2019). Varying success has been reported with duloxetine, amitriptyline, gabapentin and pregabalin in case series data (Abrams et al., 2021). In general, patients with normal skin biopsy tend to have better outcomes as compared to those with abnormal skin biopsy findings (Abrams et al., 2021). IVIG has also demonstrated considerable success (Novak, 2020; McAlpine et al., 2022). In a case series of patients with SFN in the setting of COVID-19, all three out of four patients that agreed to proceed with IVIG demonstrated significant improvement of their symptoms with one patient having complete clinical resolution (McAlpine et al., 2022). Corticosteroids are also known to be effective, especially in young patients with rapid onset SFN (Dabby et al., 2006).

#### COVID-19 vaccination and small fiber neuropathy

One case reported SNF onset 1 week post COVID-19 vaccination. Symptoms were described as subacute intense burning dysesthesias in an apparent length dependent distribution, debuting at the feet and subsequently hands. SFN was confirmed via skin biopsy (Waheed et al., 2021). There is little data at this time to support any link between COVID-19 Vaccination and SFN.

## Conclusion

In summary, peripheral neuropathies including GBS, MG and SFN can be caused or worsened by COVID-19. The incidence of severe cases has abated, in part due to a decreasing prevalence of SARS-CoV-2 infections worldwide. That being said, increased survival rates, emerging variants and the fact that vaccines are by design immunogenic (da Silva et al., 2021) signifies that a large population remains vulnerable to autoimmune mediated neurological complications of COVID-19. Thus, clinicians treating acutely ill or convalescent patients must be alert to this.

Given there is a lag between COVID-19 and symptomatic debut (Shahrizaila et al., 2021; Lee et al., 2022; Sansone and Bonifati, 2022), little can be said of treatment for concurrent COVID-19 and GBS, SFN or MG. PLEX appeared to offer the most benefit for these diseases with concomitant severe COVID-19 patients. Yet this is not standard of care and further studies are needed. Furthermore it is possible said immunomodulatory therapies will be superseded by more targeted therapies. It is possible emerging treatments for COVID-19-mediated hyperimmune cytokine response can be parlayed into novel therapies for peripheral neuropathies in the future, as appears to be the case for tocilizumab (Anand et al., 2020).

## Author contributions

EE, AM, and FG evaluated the relevancy articles. DD and KL aided in reviewing the manuscript for accuracy and editing. FG served as the final arbiter for inclusion and is cited when appropriate. FG additionally reviewed the relevant references from these publications that focused on COVID-19 pathophysiology. All authors contributed to the article and approved the submitted version.

## Conflict of interest

The authors declare that the research was conducted in the absence of any commercial or financial relationships that could be construed as a potential conflict of interest.

## Publisher’s note

All claims expressed in this article are solely those of the authors and do not necessarily represent those of their affiliated organizations, or those of the publisher, the editors and the reviewers. Any product that may be evaluated in this article, or claim that may be made by its manufacturer, is not guaranteed or endorsed by the publisher.
